# Rank-invariant estimation of inbreeding coefficients

**DOI:** 10.1038/s41437-021-00471-4

**Published:** 2021-11-25

**Authors:** Qian S. Zhang, Jérôme Goudet, Bruce S. Weir

**Affiliations:** 1 Department of Biostatistics, University of Washington, Seattle, WA 98195-1617 USA; 2 Department of Ecology and Evolution, University of Lausanne, CH-1015 Lausanne, Switzerland

**Keywords:** Population genetics, Evolutionary biology, Molecular ecology

## Abstract

The two alleles an individual carries at a locus are identical by descent (ibd) if they have descended from a single ancestral allele in a reference population, and the probability of such identity is the inbreeding coefficient of the individual. Inbreeding coefficients can be predicted from pedigrees with founders constituting the reference population, but estimation from genetic data is not possible without data from the reference population. Most inbreeding estimators that make explicit use of sample allele frequencies as estimates of allele probabilities in the reference population are confounded by average kinships with other individuals. This means that the ranking of those estimates depends on the scope of the study sample and we show the variation in rankings for common estimators applied to different subdivisions of 1000 Genomes data. Allele-sharing estimators of within-population inbreeding relative to average kinship in a study sample, however, do have invariant rankings across all studies including those individuals. They are unbiased with a large number of SNPs. We discuss how allele sharing estimates are the relevant quantities for a range of empirical applications.

## Introduction

Allelic dependence at a locus is usually quantified by inbreeding coefficients for individuals or populations, with these measures referring either to correlations of allelic state indicators (Wright, 1922) or to probabilities of identity by descent, ibd, (Malécot, 1948). Here we use ibd and we have advocated allele-sharing estimators ((Weir & Goudet, 2017), WG17 henceforth; (Goudet et al., 2018)) that are unbiased for individual and population inbreeding coefficients relative to average kinships among specified pairs of individuals. Estimators such as those in PLINK ((Purcell et al., 2007) and GCTA (Yang et al., 2011), that use sample allele frequencies, confound inbreeding estimates by the averages of individual kinships. Our work recognizes the need to estimate inbreeding coefficients from many millions of SNP genotypes where likelihood methods may not be feasible and we employ moment-based methods.

There have been many published accounts of inbreeding estimation, including the recent evaluation of several methods by Alemu et al. (2021). Among those that refer to allele sharing, Li & Horvitz (1953) discussed an inbreeding estimator based on observed homozygosity, i.e., within-individual sharing of maternal and paternal alleles. They compared observed sharing to the value expected without inbreeding. They also constructed an estimator from the proportions of each allele type that were homozygous in a sample and gave an expression that was investigated further by Ritland (1996). Ritland used allele sharing within and between individuals and his inbreeding estimates assumed “independence or near-independence” of individuals. If individuals are not independent, the rankings of his inbreeding coefficient estimates change with the sample. In WG17 we estimated inbreeding coefficients by comparing within-individual allele-sharing to average sharing between pairs of individuals in a sample. By not making explicit use of sample allele frequencies, we preserved the ranking of estimates across different samples and this is our central theme here.

Ritland’s individual-level inbreeding coefficients were also derived by Yang et al. (2011) as the correlation between uniting gametes and were expressed in terms of allele dosages for an individual and sample allele frequencies. This estimator was written as ${\hat{F}}_{{{{{{\rm{UNI}}}}}}}$ in Yengo et al. (2017), and is less biased than the estimator in Yang et al. (2011) obtained from the diagonal elements of a genomic relationship matrix (GRM) of VanRaden (2008). We compare these two estimates below with allele-sharing and other methods: pedigree-based path-counting (Wright, 1922), maximum-likelihood estimation, MLE, (e.g., (Hall et al., 2012)) and runs of homozygosity (ROH) (e.g., (Ceballos et al., 2018)).

## Methods

### Statistical sampling

We can describe the dependence between pairs of uniting alleles in a single population without invoking an evolutionary model for the history of the population. In this “statistical sampling” framework (Weir, 1996) we do not consider the variation associated with evolutionary processes but we do consider the variation among samples from the same population. Although extensive sets of genetic data allow individual-level inbreeding coefficients to be estimated with high precision, we start with population-level estimation.

Allelic dependencies can be quantified with the within-population inbreeding coefficient, written here as *f*_*W*_ to emphasize it is a within-population quantity, defined by

1 $${H}_{l}=2{p}_{l}(1-{p}_{l})(1-{f}_{W})$$

where *H*_*l*_ is the population proportion of heterozygotes for the reference allele at SNP *l* and *p*_*l*_ is the population proportion of that allele. The same value of *f*_*W*_ is assumed to apply for all SNPs. An immediate consequence of this definition is that the population proportions of homozygotes for the reference and alternative alleles are ${p}_{l}^{2}+{p}_{l}(1-{p}_{l}){f}_{W}$ and ${(1-{p}_{l})}^{2}+{p}_{l}(1-{p}_{l}){f}_{W}$ respectively. This formulation allows *f*_*W*_ to be negative, with the maximum of −*p*_*l*_/(1 − *p*_*l*_) and −(1 − *p*_*l*_)/*p*_*l*_ as lower bound. It is bounded above by 1. Hardy–Weinberg equilibrium, HWE, corresponds to *f*_*W*_ = 0 and textbooks (e.g., (Hedrick, 2000)) point out that negative values of *f*_*W*_ indicate more heterozygotes than expected under HWE.

Observed heterozygote proportions ${\tilde{H}}_{l}$ have *H*_*l*_ as within-population expectation ${{{{{{\mathcal{E}}}}}}}_{W}$ over samples from the study population, ${{{{{{\mathcal{E}}}}}}}_{W}({\tilde{H}}_{l})={H}_{l}$, and this would provide a simple estimator of *f*_*W*_ if the population allele proportions were known. In practice, however, these proportions are unknown. Steele et al. (2014) suggested use of data external to the study sample to provide reference allele proportions in forensic applications where a reference database is used for making inferences about the population relevant for a particular crime. The more usual approach is to use study sample proportions ${\tilde{p}}_{l}$ in place of the true proportions *p*_*l*_, as in equation 1 of Li & Horvitz (1953):

2 $${\hat{f}}_{{W}_{l}}=1-\frac{{\tilde{H}}_{l}}{2{\tilde{p}}_{l}(1-{\tilde{p}}_{l})}$$

The moment estimator in Eq. (2) is also an MLE of *f*_*W*_ when only one locus is considered, but it is biased (Robertson & Hill, 1984) since not only is it a ratio of statistics but also the expected value ${{{{{{\mathcal{E}}}}}}}_{W}[2{\tilde{p}}_{l}(1-{\tilde{p}}_{l})]$ over repeated samples of *n* from the population is 2*p*_*l*_(1 − *p*_*l*_)[1 − (1 + *f*_*W*_)/(2*n*)] (e.g., (Weir, 1996), p39).

This approach can be used to estimate the within-population inbreeding coefficient *f*_*j*_ for each individual *j* in a sample from one population. These are the “simple” estimators of Hall et al. (2012) and the ${\hat{f}}_{{{{{{{\rm{HOM}}}}}}}_{j}}$ of Yengo et al. (2017):

3 $${\hat{f}}_{{{{{{{\rm{HOM}}}}}}}_{jl}}=1-\frac{{\tilde{H}}_{jl}}{2{\tilde{p}}_{l}(1-{\tilde{p}}_{l})}$$

The sample heterozygosity indicator ${\tilde{H}}_{jl}$ is one if individual *j* is heterozygous at SNP *l* and is zero otherwise. Averaging Eq. (3) over individuals gives the estimator based on SNP *l* in Eq. (2).

A single SNP provides estimates that are either 1 or a negative value depending on ${\tilde{p}}_{l}$, so many SNPs are used in practice. In both Hall et al. (2012) and Yengo et al. (2017) data were combined over loci as weighted or “ratio of averages” estimators:

4 $${\hat{f}}_{{{{{{{\rm{Hom}}}}}}}_{j}}=1-\frac{{\sum }_{l}({\tilde{H}}_{jl})}{{\sum }_{l}[2{\tilde{p}}_{l}(1-{\tilde{p}}_{l})]}$$

Gazal et al. (2014) referred to this estimator as *f*_PLINK_ as it is an option in PLINK. We show below the good performance of this weighted estimator for large sample sizes and large numbers of loci. We will consider throughout that a large number *L* of SNPs are used so that ratios of sums of statistics over loci, such as in Eq. (4), have expected values equal to the ratio of expected values of their numerators and denominators. Ochoa & Storey (2021) showed statistics of the form ${\tilde{A}}_{L}/{\tilde{B}}_{L}$, where ${\tilde{A}}_{L}=\mathop{\sum }\nolimits_{l = 1}^{L}{a}_{l}/L$ and ${\tilde{B}}_{L}=\mathop{\sum }\nolimits_{l = 1}^{L}{b}_{l}/L$, have expected values that converge almost surely to the ratio *A*/*B* when ${{{{{{\mathcal{E}}}}}}}_{W}({\tilde{A}}_{L})=A{c}_{L}$ and ${{{{{{\mathcal{E}}}}}}}_{W}({\tilde{B}}_{L})=B{c}_{L}$. This result rests on the expectations ${{{{{{\mathcal{E}}}}}}}_{W}({a}_{l})=A{c}_{l}$ and ${{{{{{\mathcal{E}}}}}}}_{W}({b}_{l})=B{c}_{l}$ with ${c}_{L}=\mathop{\sum }\nolimits_{l = 1}^{L}{c}_{l}/L$. It requires ∣*a*_*l*_∣, ∣*b*_*l*_∣ to both be no greater than some finite quantity *C*, *c*_*L*_ to converge to a finite value *c* as *L* increases, and for *Bc* not to be zero. For the ratio in Eq. (4), ${a}_{l}={\tilde{H}}_{jl}$, ${b}_{l}=2{\tilde{p}}_{l}(1-{\tilde{p}}_{l})$ so *A* = (1 − *f*_*j*_), *B* = 1 for large sample sizes *n*, and *c*_*L*_ = ∑_*l*_2*p*_*l*_(1 − *p*_*l*_)/*L* ≤ 1/2. The conditions are satisfied providing at least one SNP is polymorphic. For an “average of ratios” estimator of the form $\mathop{\sum }\nolimits_{l = 1}^{L}({a}_{l}/{b}_{l})/L$, the denominators *b*_*l*_ can be very small and convergence of its expected value is not assured.

As an alternative to using sample allele frequencies, Hall et al. (2012) used maximum likelihood to estimate population allele proportions for multiple loci whereas Ayres & Balding (1998) used Markov chain Monte Carlo methods in a Bayesian approach that integrated out the allele proportion parameters. Neither of those papers considered data of the size we now face in sequence-based studies of many organisms, and we doubt the computational effort to estimate, or integrate over, hundreds of millions of allele proportions in Eqs. (2) or (4) adds much value to inferences about *f*. The allele-sharing estimators we describe below regard allele probabilities as unknown nuisance parameters and we show how to avoid estimating them or assigning them values.

Hall et al. (2012) used an EM algorithm to find MLEs for *f*_*j*_ when population allele proportions were regarded as being known and equal to sample proportions. Alternatively, a grid search can be conducted over the range of validity for the single parameter *f*_*j*_ that maximizes the log-likelihood

$${{{{\mathrm{ln}}}}}\,[{{{{{\rm{Lik}}}}}}({f}_{j})]={{{{{\rm{Constant}}}}}}+\mathop{\sum }\limits_{l=1}^{L}\{{\tilde{H}}_{jl}{{{{\mathrm{ln}}}}}\,[(1-{f}_{j})]+(1-{\tilde{H}}_{jl}){{{{\mathrm{ln}}}}}\,[1-2{\tilde{p}}_{l}(1-{\tilde{p}}_{l})(1-{f}_{j})]\}$$

Estimation of the within-population inbreeding coefficients *f*_*W*_ (*F*_*IS*_ of (Wright, 1922)) and *f*_*j*_ does not require any information beyond genotype proportions in samples from a study population, nor does it make any assumptions about that population or the evolutionary forces that shaped the population. The coefficients are simply measures of dependence of pairs of alleles within individuals.

### Genetic sampling

Inbreeding parameters of most interest in genetic studies are those that recognize the contribution of previous generations to inbreeding in the present study population. This requires accounting for “genetic sampling” (Weir, 1996) between generations, thereby leading to an ibd interpretation of inbreeding: ibd alleles descend from a single allele in a reference population. It also allows the prediction of inbreeding coefficients by path counting when pedigrees are known (Wright, 1922). If individual *J* is ancestral to both individuals $j^{\prime}$ and *j″*, and if there are *n* individuals in the pedigree path joining $j^{\prime}$ to *j″* through *J*, then *F*_*j*_ = ∑(0.5)^*n*^(1 + *F*_*J*_) where *F*_*J*_ is the inbreeding coefficient of ancestor *J* and *F*_*j*_ is the inbreeding coefficient of offspring *j* of parents $j^{\prime}$ and *j″*. The sum is over all ancestors *J* and all paths joining $j^{\prime}$ to *j″* through *J*. The expression is also the coancestry ${\theta }_{j^{\prime} j^{\prime\prime} }$ of $j^{\prime}$ and *j″*: the probability an allele drawn randomly from $j^{\prime}$ is ibd to an allele drawn randomly from *j″*.

The allele proportion *p*_*l*_ in a study population has expectation *π*_*l*_ over evolutionary replicates of the population from an ancestral reference population to the present time. Sample allele proportions ${\tilde{p}}_{l}$ provide information about the population proportions *p*_*l*_, and their statistical sampling properties follow from the binomial distribution. We do not invoke a specific genetic sampling distribution for the *p*_*l*_ about their expectations *π*_*l*_ although we do assume the second moments of that distribution depend on probabilities of ibd for pairs of alleles. One consequence of the assumed moments is that the probability of individual *j* in the study sample being heterozygous, i.e., the total expected value ${{{{{{\mathcal{E}}}}}}}_{T}$ of the heterozygosity indicator over replicates of the history of that individual, is

5 $${{{{{{\mathcal{E}}}}}}}_{T}({\tilde{H}}_{{j}_{l}})=2{\pi }_{l}(1-{\pi }_{l})(1-{F}_{j})$$

The quantity *F*_*j*_ is the individual-specific version of *F*_*IT*_ of Wright (1922) and we can regard it as the probability the two alleles at any locus for individual *j* are ibd. There is an implicit assumption in Eq. (5) that the reference population needed to define ibd is infinite and in HWE: there is probability *F*_*j*_ that *j* has homologous alleles with a single ancestral allele in that population and probability (1 − *F*_*j*_) of *j* having homologous alleles with distinct ancestral alleles there. In the first place, the single ancestral allele has probability *π* of being the reference allele for that locus and the implicit assumption is that two ancestral alleles are both the reference type with probability *π*^2^. This does not mean there is an actual ancestral population with those properties, any more than use of ${{{{{{\mathcal{E}}}}}}}_{T}$ means there are actual replicates of the history of any population or individual, and we note that Eq. (5) does not allow higher heterozygosity than predicted by HWE. Nonetheless, the concept of ibd allows theoretical constructions of great utility and we now present a framework for approaching empirical situations.

Inbreeding, or ibd, implies a common ancestral origin for uniting alleles and statements about sample allele proportions ${\tilde{p}}_{l}$ require consideration of possible ibd for other pairs of alleles in the sample. The total expectation of $2{\tilde{p}}_{l}(1-{\tilde{p}}_{l})$ over samples from the population and over evolutionary replicates of the study population is ((Weir, 1996), p176)

6 $${{{{{{\mathcal{E}}}}}}}_{T}[2{\tilde{p}}_{l}(1-{\tilde{p}}_{l})]=2{\pi }_{l}(1-{\pi }_{l})\left[(1-{\theta }_{S})-\frac{1}{2n}\left(1+{F}_{W}-2{\theta }_{S}\right)\right]$$

where *F*_*W*_ is the parametric inbreeding coefficient averaged over sample members, ${F}_{W}=\mathop{\sum }\nolimits_{j = 1}^{n}{F}_{j}/n$, and *θ*_*S*_ is the average parametric coancestry in the sample, ${\theta }_{S}=\mathop{\sum }\nolimits_{j = 1}^{n}{\sum }_{j^{\prime} \ne j}{\theta }_{jj^{\prime} }/[n(n-1)]$. Equivalent expressions were given by McPeek et al. (2004) and DeGiorgio and Rosenberg (2009). We note the relationship *f*_*W*_ = (*F*_*W*_ − *θ*_*S*_)/(1 − *θ*_*S*_) given by Wright (1922) and we showed in WG17 the equivalent expression *f*_*j*_ = (*F*_*j*_ − *θ*_*S*_)/(1 − *θ*_*S*_) for individual-specific values (*θ*_*S*_ is Wright’s *F*_*ST*_).

For a large number of SNPs, the expectation of a ratio estimator of the type considered here is the ratio of expectations (Ochoa & Storey, 2021). Therefore, the total expectations of the ${\hat{f}}_{{{{{{{\rm{Hom}}}}}}}_{j}}$, taking into account both statistical and genetic sampling, are

7 $${{{{{{\mathcal{E}}}}}}}_{T}({\hat{f}}_{{{{{{{\rm{HOM}}}}}}}_{j}})=1-\frac{1-{F}_{j}}{(1-{\theta }_{S})-\frac{1}{2n}\left(1+{F}_{W}-2{\theta }_{S}\right)}=\frac{{f}_{j}-\frac{1}{2n}(1+{f}_{W})}{1-\frac{1}{2n}(1+{f}_{W})}$$

For all sample sizes, ${\hat{f}}_{{{{{{{\rm{HOM}}}}}}}_{j}}$ has an expected value less than the true value *f*_*j*_, with the bias being of the order of 1/*n*. The ranking of ${{{{{{\mathcal{E}}}}}}}_{T}({\hat{f}}_{{{{{{{\rm{HOM}}}}}}}_{j}})$ values, however, is the same as the ranking of the *f*_*j*_ and, therefore, of the *F*_*j*_. For large sample sizes, Eq. (7) reduces to ${{{{{{\mathcal{E}}}}}}}_{T}({\hat{f}}_{{{{{{{\rm{HOM}}}}}}}_{j}})={f}_{j}$. Averaging over individuals shows that ${{{{{{\mathcal{E}}}}}}}_{T}({\hat{f}}_{{{{{{\rm{HOM}}}}}}})={f}_{W}$: the population-level estimator in Eq. (2) has total expectation of *f*_*W*_, not *F*_*W*_.

A different outcome is found for the ${\hat{f}}_{{{{{{{\rm{UNI}}}}}}}_{j}}$ estimator of Yengo et al. (2017) (i.e., ${\hat{f}}^{III}$ of Yang et al. (2011); ${\hat{f}}_{{{{{{\rm{GCTA}}}}}}3}$ of (Gazal et al., 2014)). This estimator, with the weighted (w) ratio of averages over loci we recommend, as opposed to the unweighted (u) average of ratios over loci used in their papers, is

8 $${\hat{f}}_{{{{{{{\rm{UNI}}}}}}}_{j}}^{w}=\frac{\mathop{\sum }\nolimits_{l = 1}^{L}[{X}_{jl}^{2}-(1+2{\tilde{p}}_{l}){X}_{jl}+2{\tilde{p}}_{l}^{2}]}{\mathop{\sum }\nolimits_{l = 1}^{L}2{\tilde{p}}_{l}(1-{\tilde{p}}_{l})}$$

In this equation *X*_*jl*_ is the reference allele dosage, the number of copies of the reference allele, at SNP *l* for individual *j*. It is equivalent to the estimator given by (Ritland (1996), eq. 5) and attributed by him to Li & Horvitz (1953).

Ochoa & Storey (2021) showed that ${\hat{f}}_{{{{{{{\rm{UNI}}}}}}}_{j}}^{w}$ has expectation, for a large number of SNPs and a large sample size, of

9 $${{{{{{\mathcal{E}}}}}}}_{T}({\hat{f}}_{{{{{{{\rm{UNI}}}}}}}_{j}}^{w})=\frac{{F}_{j}-2{{{\Psi }}}_{j}+{\theta }_{S}}{1-{\theta }_{S}}={f}_{j}-2{\psi }_{j}$$

where Ψ_*j*_ is the average coancestry of individual *j* with other members of the study sample: ${{{\Psi }}}_{j}=\mathop{\sum }\nolimits_{j^{\prime} = 1,j^{\prime} \ne j}^{n}{\theta }_{jj^{\prime} }/(n-1)$. We term *ψ*_*j*_ = (Ψ_*j*_ − *θ*_*S*_)/(1 − *θ*_*S*_) the within-population individual-specific average kinship coefficient. The Ψ_*j*_ have an average of *θ*_*S*_ over members of the sample, so the average of the *ψ*_*j*_’s is zero and expected value of the average of the ${\hat{f}}_{{{{{{{\rm{UNI}}}}}}}_{j}}^{w}$ is *f*_*W*_, as is the case for ${\hat{f}}_{{{{{{{\rm{AS}}}}}}}_{j}}$ below.

Equation (9) shows that the ${\hat{f}}_{{{{{{{\rm{UNI}}}}}}}_{j}}^{w}$ have expected values with the same ranking as the *F*_*j*_ values only if every individual *j* in the sample has the same average kinship *ψ*_*j*_ with other sample members.

Finally, we mention another common estimator described by VanRaden (2008), termed *f*_GCTA1_ by Gazal et al. (2014) and available from the GCTA software (Yang et al., 2011) with option --ibc. We referred to this as the “standard” estimator in WG17. The weighted version for multiple loci is

10 $${\hat{f}}_{{{{{{{\rm{STD}}}}}}}_{j}}^{w}=\frac{{\sum }_{l}{({X}_{jl}-2{\tilde{p}}_{l})}^{2}}{{\sum }_{l}2{\tilde{p}}_{l}(1-{\tilde{p}}_{l})}-1$$

and it has the large-sample expectation of (*f*_*j*_ − 4*ψ*_*j*_) as is implied by WG17 (Eq. 13) and as was given by Ochoa & Storey (2021). We summarize the various measures of inbreeding and coancestry in Table 1, and we include sample sizes in the expectations shown in Table 2.

**Table 1:** Measures of inbreeding and coancestry.

Measure	Description	Evaluation
*F*_*j*_	Inbreeding coefficient for individual *j*:	*F*_PED_: Path counting.
	ibd probability for homologous alleles	*F*_Gold_: Actual ibd in simulations.
${\theta }_{jj^{\prime} }$	Coancestry for individuals $j,j^{\prime}$: ibd probability	*θ*_PED_: Path counting.
	for random alleles from *j* and $j^{\prime}$.	*θ*_Gold_: Actual ibd in simulations.
The following hold for PED and Gold values.		
*F*_*W*_	Average inbreeding coefficient.	${F}_{W}=\frac{1}{n}\mathop{\sum }\nolimits_{j = 1}^{n}{F}_{j}$ for *n* individuals.
Ψ_*j*_	Average coancestry coefficient for individual *j*.	${{{\Psi }}}_{j}=\frac{1}{n-1}{\mathop{\sum }\nolimits_{j^{\prime} = 1}^{n}}_{j^{\prime} \ne j}{\theta }_{jj^{\prime} }$
*θ*_*S*_	Average coancestry coefficient.	${\theta }_{S}=\frac{1}{n}\mathop{\sum }\nolimits_{j = 1}^{n}{{{\Psi }}}_{j}$
*f*_*j*_	Within-population inbreeding coefficient	${f}_{j}=\frac{{F}_{j}-{\theta }_{S}}{1-{\theta }_{S}}$
	for individual *j*.	
*f*_*W*_	Average within-population inbreeding coefficient.	${f}_{W}=\frac{{F}_{W}-{\theta }_{S}}{1-{\theta }_{S}}$
*ψ*_*j*_	Within-population average kinship coefficient for	${\psi }_{j}=\frac{{{{\Psi }}}_{j}-{\theta }_{S}}{1-{\theta }_{S}}$
	individual *j*.

**Table 2:** Estimators of inbreeding.

Estimate	Calculation^a^	Expected Value^b^
${\hat{F}}_{{{{{{{\rm{ROH}}}}}}}_{j}}$	Proportion of homozygous blocks.	No explicit expression.
${\hat{f}}_{{{{{{{\rm{MLE}}}}}}}_{j}}$	Maximization of likelihood for *f*_*j*_.	No explicit expression.
${\hat{f}}_{{{{{{{\rm{HOM}}}}}}}_{j}}$	$1-\frac{{\sum }_{l}{X}_{jl}(2-{X}_{jl})}{{\sum }_{l}2{\tilde{p}}_{l}(1-{\tilde{p}}_{l})}$	$\frac{{f}_{j}-\frac{1}{2n}(1+{f}_{W})}{1-\frac{1}{2n}(1+{f}_{W})}$
${\hat{f}}_{{{{{{{\rm{HOM}}}}}}}_{W}}$	$1-\frac{1}{n}\mathop{\sum }\nolimits_{j = 1}^{n}\frac{{\sum }_{l}{X}_{jl}(2-{X}_{jl})}{{\sum }_{l}2{\tilde{p}}_{l}(1-{\tilde{p}}_{l})}$	$\frac{{f}_{W}-\frac{1}{2n}(1+{f}_{W})}{1-\frac{1}{2n}(1+{f}_{W})}$
${\hat{f}}_{{{{{{{\rm{AS}}}}}}}_{j}}$	$\frac{{\sum }_{l}({\tilde{A}}_{jl}-{\tilde{A}}_{Sl})}{{\sum }_{l}(1-{\tilde{A}}_{Sl})}$	*f*_*j*_
${\hat{f}}_{{{{{{{\rm{AS}}}}}}}_{W}}$	$\frac{1}{n}\mathop{\sum }\nolimits_{j = 1}^{n}{\hat{f}}_{{{{{{{\rm{AS}}}}}}}_{j}}$	*f*_*W*_
${\hat{f}}_{{{{{{{\rm{UNI}}}}}}}_{j}}^{w}$	$\frac{{\sum }_{l}[{X}_{jl}^{2}-(1+2{\tilde{p}}_{l}){X}_{jl}+2{\tilde{p}}_{l}^{2}]}{{\sum }_{l}2{\tilde{p}}_{l}(1-{\tilde{p}}_{l})}$	$\frac{{f}_{j}-2{\psi }_{j}-\frac{1}{2n}(3+4{f}_{j}-8{\psi }_{j}-{f}_{W})}{1-\frac{1}{2n}(1+{f}_{W})}$
${\hat{f}}_{{{{{{{\rm{UNI}}}}}}}_{W}}^{w}$	$\frac{1}{n}\mathop{\sum }\nolimits_{j = 1}^{n}{\hat{f}}_{{{{{{{\rm{UNI}}}}}}}_{j}}^{w}$	$\frac{{f}_{W}-\frac{3}{2n}(1+{f}_{W})}{1-\frac{1}{2n}(1+{f}_{W})}$
${\hat{f}}_{{{{{{{\rm{UNI}}}}}}}_{j}}^{u}$	$\frac{1}{L}\mathop{\sum }\nolimits_{l = 1}^{L}\frac{{X}_{jl}^{2}-(1+2{\tilde{p}}_{l}){X}_{jl}+2{\tilde{p}}_{l}^{2}}{2{\tilde{p}}_{l}(1-{\tilde{p}}_{l})}$	No explicit expression.
${\hat{f}}_{{{{{{{\rm{STD}}}}}}}_{j}}^{w}$	$\frac{{\sum }_{l}{({X}_{jl}-2{\tilde{p}}_{l})}^{2}}{{\sum }_{l}2{\tilde{p}}_{l}(1-{\tilde{p}}_{l})}-1$	$\frac{{f}_{j}-4{\psi }_{j}-\frac{1}{2n}(3+4{f}_{j}-8{\psi }_{j}-{f}_{W})}{1-\frac{1}{2n}(1+{f}_{W})}$
${\hat{f}}_{{{{{{{\rm{STD}}}}}}}_{W}}^{w}$	$\frac{1}{n}\mathop{\sum }\nolimits_{j = 1}^{n}{\hat{f}}_{{{{{{{\rm{STD}}}}}}}_{j}}^{w}$	$\frac{{f}_{W}-\frac{3}{2n}(1+{f}_{W})}{1-\frac{1}{2n}(1+{f}_{W})}$
${\hat{f}}_{{{{{{{\rm{STD}}}}}}}_{j}}^{u}$	$\frac{1}{L}\mathop{\sum }\nolimits_{l = 1}^{L}\frac{{({X}_{jl}-2{\tilde{p}}_{l})}^{2}}{2{\tilde{p}}_{l}(1-{\tilde{p}}_{l})}-1$	No explicit expression.

^a^*X*_*jl*_ is the reference allele dosage for SNP *l* in individual *j*.

^a^${\tilde{p}}_{l}=\frac{1}{2n}\mathop{\sum }\nolimits_{j = 1}^{n}{X}_{jl}$ is the sample allele frequency for SNP *l*.

^b^For weighted averages over large numbers of loci.

The ${\hat{f}}_{{{{{{\rm{HOM}}}}}}}$, ${\hat{f}}_{{{{{{\rm{UNI}}}}}}},{\hat{f}}_{{{{{{\rm{STD}}}}}}}$ and ${\hat{f}}_{{{{{{\rm{MLE}}}}}}}$ estimators of individual or population inbreeding coefficients make explicit use of sample allele proportions. This means that all four have small-sample biases, and none of the four provide estimates of the ibd quantities *F* or *F*_*j*_. We showed that ${\hat{f}}_{{{{{{\rm{HOM}}}}}}}$ is actually estimating the within-population inbreeding coefficients: the total inbreeding coefficients *relative to* the average coancestry of pairs of individuals in the sample, but ${\hat{f}}_{{{{{{\rm{UNI}}}}}}}$ and ${\hat{f}}_{{{{{{\rm{STD}}}}}}}$ are estimating expressions that also involve average kinships *ψ*.

### Allele sharing

In a genetic sampling framework, and with the ibd viewpoint, we consider within-individual allele sharing proportions *A*_*jl*_ for SNP *l* in individual *j* (we wrote *M* rather than *A* in WG17 and in (Goudet et al., 2018)). These equal one for homozygotes and zero for heterozygotes and sample values can be expressed in terms of allele dosages, ${\tilde{A}}_{jl}={({X}_{jl}-1)}^{2}$. We also consider between-individual sharing proportions ${A}_{jj^{\prime} l}$ for SNP *l* and individuals *j* and $j^{\prime}$. These are equal to one for both individuals being the same homozygote, zero for different homozygotes, and 0.5 otherwise. Observed values can be written as ${\tilde{A}}_{jj^{\prime} l}=[1+({X}_{jl}-1)({X}_{j^{\prime} l}-1)]/2$, with an average over all pairs of distinct individuals in a sample of ${\tilde{A}}_{Sl}$. Astle & Balding (2009) introduced ${\tilde{A}}_{jj^{\prime} l}$ as a measure of identity in state of alleles chosen randomly from individuals *j* and $j^{\prime}$, and Ochoa & Storey (2021) used a simple transformation of this quantity. The allele sharing for an individual with itself is *A*_*jjl*_ = (1 + *A*_*jl*_)/2.

The same logic that led to Eq. (5) provides total expectations for allele-sharing proportions for all $j,j^{\prime}$:

$$\begin{array}{lll}{{{{{{\mathcal{E}}}}}}}_{T}({\tilde{A}}_{jj^{\prime} l})&=&1-2{\pi }_{l}(1-{\pi }_{l})(1-{\theta }_{jj^{\prime} })\\ {{{{{{\mathcal{E}}}}}}}_{T}({\tilde{A}}_{Sl})&=&1-2{\pi }_{l}(1-{\pi }_{l})(1-{\theta }_{S})\end{array}$$

Note that *θ*_*jj*_ = (1 + *F*_*j*_)/2. The nuisance parameter 2*π*_*l*_(1 − *π*_*l*_) cancels out of the ratio ${{{{{{\mathcal{E}}}}}}}_{T}({\tilde{A}}_{jj^{\prime} l}-{\tilde{A}}_{Sl})/{{{{{{\mathcal{E}}}}}}}_{T}(1-{\tilde{A}}_{Sl})$ and this motivates definitions of allele-sharing estimators of the inbreeding coefficient for individual *j* and the kinship coefficient for individuals $j,j^{\prime}$ as

11 $${\hat{f}}_{{{{{{{\rm{AS}}}}}}}_{j}}=\frac{{\sum }_{l}({\tilde{A}}_{{j}_{l}}-{\tilde{A}}_{{S}_{l}})}{{\sum }_{l}(1-{\tilde{A}}_{Sl})},{\hat{\psi }}_{{{{{{{\rm{AS}}}}}}}_{jj^{\prime} }}=\frac{{\sum }_{l}({\tilde{A}}_{jj^{\prime} l}-{\tilde{A}}_{{S}_{l}})}{{\sum }_{l}(1-{\tilde{A}}_{Sl})}$$

For a large number of SNPs, these are unbiased for *f*_*j*_ and ${\psi }_{jj^{\prime} }$ for all sample sizes. We showed in WG17 there is no need to filter on minor allele frequency to preserve the lack of bias. Note that ${\hat{f}}_{{{{{{{\rm{AS}}}}}}}_{j}}$ is a linear function of the form ${a}_{S}+{b}_{S}{\tilde{A}}_{j}$ with ${\tilde{A}}_{j}$ being the total homozygosity for *j* and constants *a*_*S*_, *b*_*S*_ being the same for all individuals *j*. Changing the scope of the study, from population to world for example, preserves linearity (with different values of *a*_*S*_, *b*_*S*_). The changed estimates are linear functions of the old estimates: old and new estimates are completely correlated and are rank invariant over all samples that include particular individuals, i.e., over all reference populations. Unlike the case for ${\hat{f}}_{{{{{{\rm{UNI}}}}}}}$ or ${\hat{f}}_{{{{{{\rm{STD}}}}}}}$, rank invariance is guaranteed for ${\hat{f}}_{{{{{{{\rm{AS}}}}}}}_{j}}$ for any two individuals even if only one more individual is added to the study.

For large sample sizes, $(1-{\tilde{A}}_{Sl})\approx 2{\tilde{p}}_{l}(1-{\tilde{p}}_{l})$. Under that approximation, ${\hat{f}}_{{{{{{{\rm{AS}}}}}}}_{j}}$ is the same as ${\hat{f}}_{{{{{{{\rm{Hom}}}}}}}_{j}}$ but the approximation is not necessary in computer-based analyses. Summing the large-sample estimates over individuals not equal to *j* gives an estimator for the average individual kinship *ψ*_*j*_:

12 $${\hat{\psi }}_{{{{{{{\rm{AS}}}}}}}_{j}}=-\frac{{\sum }_{l}({X}_{jl}-2{\tilde{p}}_{l})(1-2{\tilde{p}}_{l})}{{\sum }_{l}4{\tilde{p}}_{l}(1-{\tilde{p}}_{l})}$$

Adding $2{\hat{\psi }}_{{{{{{{\rm{AS}}}}}}}_{j}}$ to ${\hat{f}}_{{{{{{{\rm{UNI}}}}}}}_{j}}^{w}$ gives ${\hat{f}}_{{{{{{{\rm{AS}}}}}}}_{j}}$, as expected, as does adding $4{\hat{\psi }}_{{{{{{{\rm{AS}}}}}}}_{j}}$ to ${\hat{f}}_{{{{{{{\rm{STD}}}}}}}_{j}}^{w}$. Similarly, ${\hat{\psi }}_{{{{{{{\rm{AS}}}}}}}_{jj^{\prime} }}$ is obtained by adding ${\hat{\psi }}_{{{{{{{\rm{AS}}}}}}}_{j}}$ and ${\hat{\psi }}_{{{{{{{\rm{AS}}}}}}}_{j^{\prime} }}$ to ${\hat{\psi }}_{{{{{{{\rm{STD}}}}}}}_{jj^{\prime} }}$, where (Yang et al., 2011)

$${\hat{\psi }}_{{{{{{{\rm{STD}}}}}}}_{jj^{\prime} }}=\frac{\mathop{\sum}\nolimits_{l}({X}_{jl}-2{\tilde{p}}_{l})({X}_{j^{\prime} l}-2{\tilde{p}}_{l})}{\mathop{\sum}\nolimits_{l}4{\tilde{p}}_{l}(1-{\tilde{p}}_{l})}$$

These are the elements of the first method for constructing the GRM given by VanRaden (2008).

When inbreeding and coancestry coefficients are defined as ibd probabilities they are non-negative, but the within-population values *f* and *ψ* will be negative for individuals, or pairs of individuals, having smaller ibd allele probabilities than do pairs of individuals in the sample, on average. Individual-specific values of *f* always have the same ranking as the individual-specific *F* values, and they are estimable. Negative estimates can be avoided by the transformation to $({\hat{f}}_{{{{{{{\rm{AS}}}}}}}_{j}}-{\hat{f}}_{{{{{{{\rm{AS}}}}}}}_{j}}^{\min })/(1-{\hat{f}}_{{{{{{{\rm{AS}}}}}}}_{j}}^{\min })$ where ${\hat{f}}_{{{{{{{\rm{AS}}}}}}}_{j}}^{\min }$ is the smallest value over individuals of the ${\hat{f}}_{{{{{{{\rm{AS}}}}}}}_{j}}$’s. We don’t see the need for this transformation, and we noted above the recognition of the utility of negative values. Ochoa & Storey (2021) wished to estimate *F*_*j*_ rather than *f*_*j*_ and, to overcome the lack of information about the ancestral population serving as a reference point for ibd, they assumed the least related pair of individuals in a sample have a coancestry of zero. We showed in WG17 that this brings estimates in line with path-counting predicted values when founders are assumed to be not inbred and unrelated, but we prefer to avoid the assumption. We stress that, absent external information or assumptions, *F* is not estimable. Instead, linear functions of *F* that describe ibd of target pairs of alleles relative to ibd in a specified set of alleles are estimable and have utility in empirical studies.

### Runs of homozygosity

Each of the inbreeding estimators considered so far has been constructed for individual SNPs and then combined over SNPs. Observed values of allelic state are used to make inferences about the unobserved state of identity by descent. Estimators based on ROH, however, suppose that ibd for a region of the genome can be observed. Although *F* is the probability an individual has ibd alleles at any single SNP, in fact ibd occurs in blocks within which there has been no recombination in the paths of descent from common ancestor to the individual’s parents. Whereas a single SNP can be homozygous without the two alleles being ibd, if many adjacent SNPs are homozygous the most likely explanation is that they are in a block of ibd (Gibson et al., 2006). There can be exceptions, from mutation for example, and several publications give strategies for identifying runs of homozygotes for which ibd may be assumed (e.g., Gazal et al. (2014); (Joshi et al., 2015)). These strategies include adjusting the size of the blocks, the numbers of heterozygotes or missing values allowed per block, the minor allele frequency, and so on. These software parameters affect the size of the estimates (Meyermans et al., 2020). Some methods (e.g., Gazal et al. (2014); (Narasimhan et al., 2016)) use hidden Markov models where ibd is the hidden status of an observed homozygote. Model-based approaches necessarily have assumptions, such as HWE in the sampled population.

We provide more details elsewhere, but we note here that ROH methods offer a useful alternative to SNP-by-SNP methods even though they cannot completely compensate for lack of information on the ibd reference population. We note also that shorter runs of ibd result from more distant relatedness of an individual’s parents, and ROH procedures can be set to distinguish recent (familial) ibd from distant (evolutionary) ibd. SNP-by-SNP estimators do not make a distinction between these two time scales.

## Results

### Simulation study

We used the quantiNemo software (Neuenschwander et al., 2019) to simulate a five-generation pedigree of hermaphroditic individuals mating randomly, excluding selfing, with each mating producing a number of offspring drawn from a Poisson distribution with mean two. The zero-th generation was made of 50 founders, the first generation had 47 individuals and the second, third, fourth and fifth generations had 58, 56, 57, and 65 individuals respectively. This pedigree was then fed to a custom R script to draw gametes from each parent at each reproductive event, allowing for recombination based on a 20 Morgan recombination map with a genetic marker every 0.1 cM, for a total of 20,000 markers.

Each of the 100 alleles per marker among the 50 founders was given a unique identifier so that alleles in subsequent generations with the same identifier had actual identity by descent relative to the founders. The average actual ibd proportions over loci, within individuals and between each pair of individuals, provided “gold standard” inbreeding and coancestry coefficients, as opposed to the pedigree-based values we calculated by path counting. The gold values for inbreeding coefficients *F*_*j*_ and coancestry coefficients ${\theta }_{jj^{\prime} }$ then allow calculation of gold values for *f*_*j*_, *ψ*_*j*_ and, therefore, ${f}_{{{{{{{\rm{STD}}}}}}}_{j}}$ and ${f}_{{{{{{{\rm{UNI}}}}}}}_{j}}$.

Finally, the two unique identifiers for each marker of the 50 founders were mapped to the SNP genotypes of the 50 founders generated with the msprime program (Kelleher et al., 2016) as follows: we assume the founders originated from a population with effective size *N*_*e*_ = 10^4^, mutation rate *μ* = 10^−9^, recombination rate between neighboring base pairs *r* = 10^−7^. We assumed 20 chromosomes each 10 Megabase (10^7^) long. The necessary arguments are mspms 100 20 -t 400 -r 40000 10000000 -p 9. This generated a dataset of 100 gametes and over 40,000 SNPs, with the first 20,000 used for the mapping of unique identifiers to SNP alleles. This mapping was applied to the genotypes of the non-founder individuals of the pedigree to generate their SNP genotypes.

The pedigree was constructed to provide fairly high levels of predicted coancestry among pairs of the 283 non-founder individuals, ranging from 0 to 0.464, with a mean of *θ*_*S*_ = 0.053, assuming the 50 founders were unrelated and not inbred. The pedigree inbreeding coefficients ranged from 0 to 0.367, with a mean of *F*_*W*_ = 0.050. The within-population inbreeding coefficient for the set of 283 non-founder individuals is *f* = (*F*_*W*_ − *θ*_*S*_)/(1 − *θ*_*S*_) = −0.003. Note, however, that the 50 individuals regarded as founders for the subsequent 283 had their own joint histories from the msprime simulation. These 50 had an average within-individual allele sharing of ${\tilde{A}}_{W}=0.80385$ and an average between-individual allele sharing of ${\tilde{A}}_{S}=0.80355$. The difference of these two proportions, which would be zero for a reference set of non-inbred and unrelated individuals, provides a within-founder allele-sharing inbreeding coefficient ${\hat{f}}_{{{{{{\rm{W}}}}}}}$ of 0.0015.

The various estimators of inbreeding examined with these data are shown in Table 2, and the correlation coefficients for each pair of estimates over the whole set of 283 non-founder individuals are shown in Table 3. There are very high correlations between pedigree and gold-standard values and also very high correlations between ${\hat{f}}_{{{{{{\rm{HOM}}}}}}}$ and ${\hat{f}}_{{{{{{\rm{AS}}}}}}}$ values, both as expected. There are lower correlations of ${\hat{f}}_{{{{{{\rm{UNI}}}}}}}$ and ${\hat{f}}_{{{{{{\rm{STD}}}}}}}$ with pedigree-based or gold-standard inbreeding coefficients since those estimates reflect both *f* and *ψ*.

**Table 3:** Correlations among inbreeding measures^a^ for simulated data.

	*F*_PED_	*F*_Gold_	${\hat{F}}_{{{{{{\rm{ROH}}}}}}}$	*f*_PED_	*f*_Gold_	${\hat{f}}_{{{{{{\rm{AS}}}}}}}$	${\hat{f}}_{{{{{{\rm{HOM}}}}}}}$	${\hat{f}}_{{{{{{\rm{MLE}}}}}}}$	${f}_{{{{{{\rm{UNI}}}}}}}^{{{{{{\rm{Gold}}}}}}}$	${\hat{f}}_{{{{{{\rm{UNI}}}}}}}^{{{{{{\rm{w}}}}}}}$	${\hat{f}}_{{{{{{\rm{UNI}}}}}}}^{{{{{{\rm{u}}}}}}}$	${f}_{{{{{{\rm{STD}}}}}}}^{{{{{{\rm{Gold}}}}}}}$	${\hat{f}}_{{{{{{\rm{STD}}}}}}}^{{{{{{\rm{w}}}}}}}$	${\hat{f}}_{{{{{{\rm{STD}}}}}}}^{{{{{{\rm{u}}}}}}}$
*F*_PED_	1.00	0.94	0.92	1.00	0.94	0.84	0.84	0.80	0.80	0.71	0.74	0.44	0.36	−0.25
*F*_Gold_	0.94	1.00	0.99	0.94	1.00	0.90	0.90	0.88	0.86	0.78	0.80	0.48	0.41	−0.24
${\hat{F}}_{{{{{{\rm{ROH}}}}}}}$	0.92	0.99	1.00	0.92	0.99	0.91	0.91	0.89	0.87	0.80	0.82	0.50	0.45	−0.20
*f*_PED_	1.00	0.94	0.92	1.00	0.94	0.84	0.84	0.80	0.80	0.71	0.74	0.44	0.36	−0.25
*f*_Gold_	0.94	1.00	0.99	0.94	1.00	0.90	0.90	0.88	0.86	0.78	0.80	0.48	0.41	−0.24
${\hat{f}}_{{{{{{\rm{AS}}}}}}}$	0.84	0.90	0.91	0.84	0.90	1.00	1.00	0.99	0.77	0.86	0.86	0.42	0.44	−0.22
${\hat{f}}_{{{{{{\rm{HOM}}}}}}}$	0.84	0.90	0.91	0.84	0.90	1.00	1.00	0.99	0.77	0.86	0.86	0.42	0.44	−0.22
${\hat{f}}_{{{{{{\rm{MLE}}}}}}}$	0.80	0.88	0.89	0.80	0.88	0.99	0.99	1.00	0.82	0.92	0.91	0.53	0.57	−0.10
${f}_{{{{{{\rm{UNI}}}}}}}^{{{{{{\rm{Gold}}}}}}}$	0.80	0.86	0.87	0.80	0.86	0.77	0.77	0.82	1.00	0.89	0.91	0.86	0.74	0.18
${\hat{f}}_{{{{{{\rm{UNI}}}}}}}^{{{{{{\rm{w}}}}}}}$	0.71	0.78	0.80	0.71	0.78	0.86	0.86	0.92	0.89	1.00	0.98	0.75	0.84	0.17
${\hat{f}}_{{{{{{\rm{UNI}}}}}}}^{{{{{{\rm{u}}}}}}}$	0.74	0.80	0.82	0.74	0.80	0.86	0.86	0.91	0.91	0.98	1.00	0.76	0.80	0.17
${f}_{{{{{{\rm{STD}}}}}}}^{{{{{{\rm{Gold}}}}}}}$	0.44	0.48	0.50	0.44	0.48	0.42	0.42	0.53	0.86	0.75	0.76	1.00	0.87	0.55
${\hat{f}}_{{{{{{\rm{STD}}}}}}}^{{{{{{\rm{w}}}}}}}$	0.36	0.41	0.45	0.36	0.41	0.44	0.44	0.57	0.74	0.84	0.80	0.87	1.00	0.53
${\hat{f}}_{{{{{{\rm{STD}}}}}}}^{{{{{{\rm{u}}}}}}}$	−0.25	−0.24	−0.20	−0.25	−0.24	−0.22	−0.22	−0.10	0.18	0.17	0.17	0.55	0.53	1.00

^a^As shown in Tables 1 and 2.

We see in Table 3 that ${\hat{F}}_{{{{{{\rm{ROH}}}}}}}$ values are the most highly correlated with *F*_Gold_: this high correlation was obtained by adjusting the block size (100 SNPs) and the block overlap amount (50 SNPs) to bring estimates close to the known *F*_Gold_ values. In practice the *F*_Gold_ values are not known and the other estimators are all evaluated without external information. The high correlation of ${\hat{f}}_{{{{{{\rm{AS}}}}}}}$ and maximum likelihood values suggests that ${\hat{f}}_{{{{{{\rm{MLE}}}}}}}$ is estimating *f* rather than *F* because it uses the sample allele frequencies in place of the unknown allele probabilities. The weighted and unweighted versions of ${\hat{f}}_{{{{{{\rm{UNI}}}}}}}$ are highly correlated with each other and with their gold values, but not with *f*_Gold_. There are generally low correlations for weighted and unweighted ${\hat{f}}_{{{{{{\rm{STD}}}}}}}$ values.

**Fig. 1:**
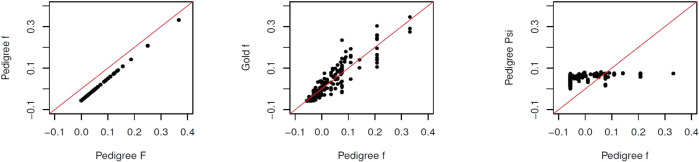
Allele sharing estimates for 283 non-founders in simulated pedigree. Left: Pedigree *f* vs Pedigree *F*; Center: Gold *f* vs Pedigree *f*; Right: Pedigree coancestry vs Pedigree *f*.

Figure 1 (left) illustrates the linear relationship between ${f}_{{{{{{{\rm{Ped}}}}}}}_{j}}$ and ${F}_{{{{{{{\rm{Ped}}}}}}}_{j}}$: ${f}_{{{{{{{\rm{Ped}}}}}}}_{j}}=({F}_{{{{{{{\rm{Ped}}}}}}}_{j}}-{\theta }_{{{{{{{\rm{Ped}}}}}}}_{S}})/(1-{\theta }_{{{{{{{\rm{Ped}}}}}}}_{S}})$ where ${\theta }_{{{{{{{\rm{Ped}}}}}}}_{S}}=0.053$ is the average coancestry of pairs of non-founders, calculated from the pedigree. The ${F}_{{{{{{{\rm{Gold}}}}}}}_{j}}$ and ${f}_{{{{{{{\rm{Gold}}}}}}}_{j}}$ values are also correlated with the corresponding pedigree values, as is shown for ${f}_{{{{{{{\rm{Gold}}}}}}}_{j}}$ in Fig. 1 (center). The variation we see in Fig. 1 (center) for ${f}_{{{{{{{\rm{Gold}}}}}}}_{j}}$ around ${F}_{{{{{{{\rm{Ped}}}}}}}_{j}}$ reflects the variation of actual inbreeding about expected values, even for whole genomes, pointed out by Hill & Weir (2011). Wang (2016) showed that the number of SNPs also has an effect. The lack of relationship between pedigree-based values of individual average coancestry *ψ*_*j*_ and individual inbreeding *f*_*j*_, leading to variable rankings for some estimators based on sample allele frequencies, is shown in Fig. 1 (right).

**Fig. 2:**
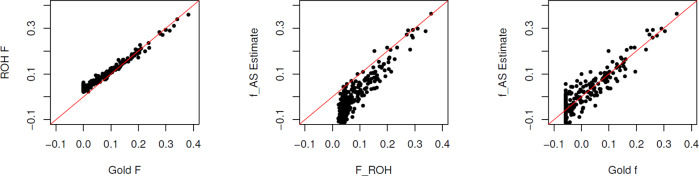
Values of ROH estimates of *F* and allele-sharing estimates of *f* for 283 non-founders in simulated pedigree. Left: ${\hat{F}}_{{{{{{\rm{ROH}}}}}}}$ vs *F*_Gold_; Center: ${\hat{f}}_{{{{{{\rm{AS}}}}}}}$ vs ${\hat{F}}_{{{{{{\rm{ROH}}}}}}}$; Right: ${\hat{f}}_{{{{{{\rm{AS}}}}}}}$ vs *f*_Gold_.

**Fig. 3:**
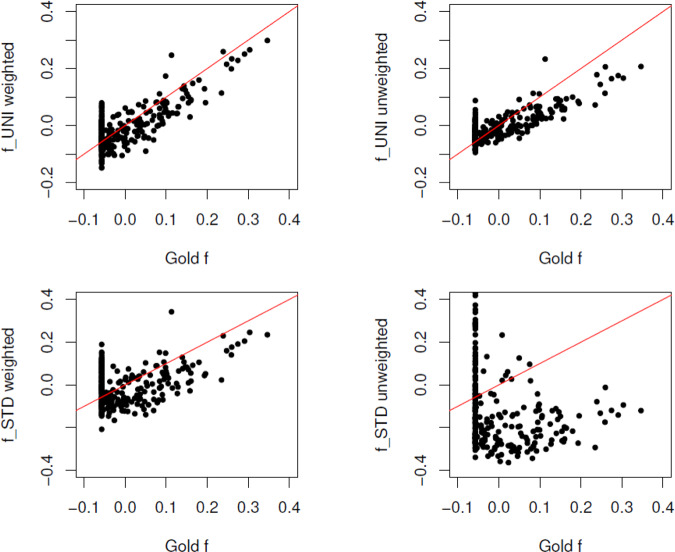
Values of UNI and STD estimates for 283 non-founders in simulated pedigree. Top left: ${\hat{f}}_{{{{{{\rm{UNI}}}}}}}^{w}$ vs ${f}_{{{{{{{\rm{Gold}}}}}}}_{j}}$; Top right: ${\hat{f}}_{{{{{{\rm{STD}}}}}}}^{w}$ vs ${f}_{{{{{{{\rm{Gold}}}}}}}_{j}}$; Bottom left: ${\hat{f}}_{{{{{{\rm{UNI}}}}}}}^{u}$ vs ${f}_{{{{{{{\rm{Gold}}}}}}}_{j}}$; Bottom right: ${\hat{f}}_{{{{{{\rm{STD}}}}}}}^{u}$ vs ${f}_{{{{{{{\rm{Gold}}}}}}}_{j}}$.

Figure 2 (left) illustrates the similarity of ${\hat{F}}_{{{{{{\rm{ROH}}}}}}}$ and *F*_Gold_ and Fig. 2 (center) shows general agreement between ${\hat{F}}_{{{{{{\rm{ROH}}}}}}}$ and ${\hat{f}}_{{{{{{\rm{AS}}}}}}}$, bearing in mind that ${\hat{f}}_{{{{{{\rm{AS}}}}}}}$ estimates (*F* − *θ*_*S*_)/(1 − *θ*_*S*_). Figure 2 (right) shows general agreement of the allele-sharing estimators ${\hat{f}}_{{{{{{{\rm{AS}}}}}}}_{j}}$ with the gold-standard within-population inbreeding coefficients ${f}_{{{{{{{\rm{Gold}}}}}}}_{j}}$. Figure 3 shows ${\hat{f}}_{{{{{{{\rm{UNI}}}}}}}_{j}}$ to be a better estimator of ${f}_{{{{{{{\rm{Gold}}}}}}}_{j}}$ than is ${\hat{f}}_{{{{{{{\rm{STD}}}}}}}_{j}}$, as noted by Yang et al. (2011), and better performance for the weighted than unweighted averages over SNPs but still not as good as ${\hat{f}}_{{{{{{{\rm{AS}}}}}}}_{j}}$.

### 1000 genomes data

**Fig. 4:**
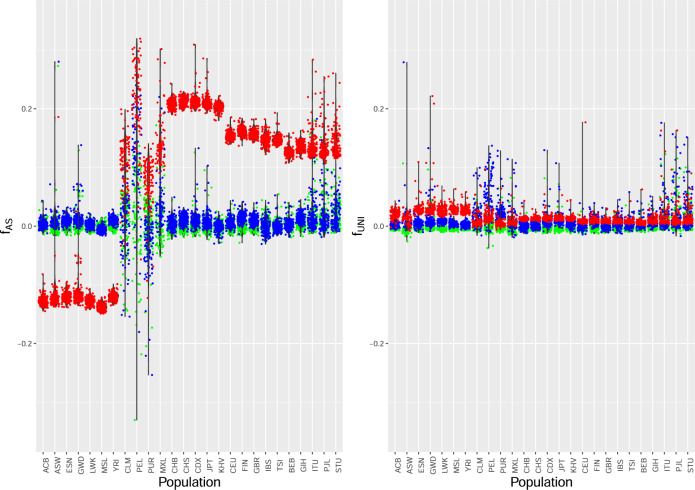
Individual inbreeding coefficient estimates for 1000 Genomes data. Left panel: ${\hat{f}}_{{{{{{\rm{AS}}}}}}}$; Right panel: ${\hat{f}}_{{{{{{\rm{UNI}}}}}}}^{u}$. Green: Population as reference; Blue: Continental group as reference; Red: World as reference. Populations, left to right: (AFR) ACB, ASW, ESN, GWD, LWK, MSL, YRI; (AMR) CLM, PEL, PUR, MXL; (EAS) CHB, CHS, CDX, JPT, KHV; (EUR) CEU, FIN, GBR, IBS, TSI; (SAS) BEB, GIH, ITU, PJL, STU.

We used 77m SNPs from the 22 autosomes for the 26 populations of the 1000 Genomes whole genome data to estimate inbreeding coefficients for all 2504 individuals in the project. Our focus was on the algebraic invariance of estimate rankings as the reference set of individuals changed from the population from which each individual was sampled, to the continental group for that population, to the whole world. We calculated the estimates ${\hat{f}}_{{{{{{{\rm{AS}}}}}}}_{j}}$ and ${\hat{f}}_{{{{{{{\rm{UNI}}}}}}}_{j}}^{u}$ for each individual and each reference set, and ranked estimates within each population. The two sets of estimates for all individuals are shown separately in Fig. 4. Figures S1 and S2 show ${\hat{f}}_{{{{{{{\rm{UNI}}}}}}}_{j}}^{u}$ vs ${\hat{f}}_{{{{{{{\rm{AS}}}}}}}_{j}}$ for estimates and ranks respectively.

**Fig. 5:**
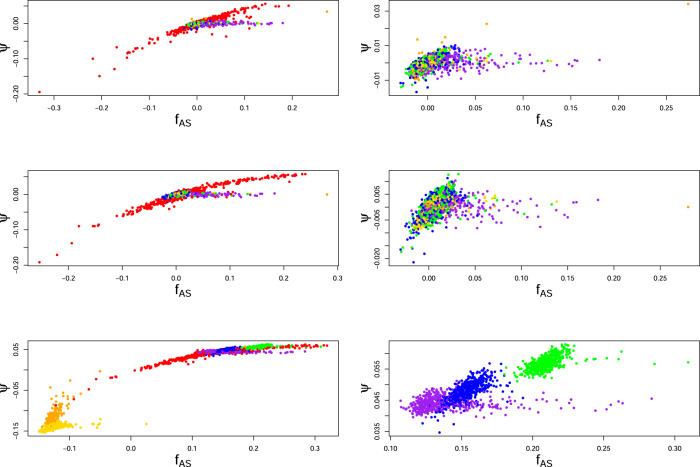
Estimates of within-population individual-specific average kinships vs estimates of within-population individual-specific inbreeding coefficients for 1000 Genomes data. *Y*-axis: ${\hat{\psi }}_{{{{{{{\rm{AS}}}}}}}_{j}}$; *X*-axis: ${\hat{f}}_{{{{{{{\rm{AS}}}}}}}_{j}}$. Top: Population as reference set; Center: Continent as reference set; Bottom: World as reference set. Left: All populations; Right: Excluding AMR populations in top and center rows. Excluding AMR and AFR in bottom row. Gold: AFR (not ACB or ASW); Orange: AFR (ACB and ASW); Red: AMR; Purple: SAS; Blue: EUR; Green: EAS.

Figure 4 shows that within-population inbreeding coefficients ${\hat{f}}_{{{{{{\rm{AS}}}}}}}$ for all 1000 Genomes populations outside the AMR group are essentially the same, and generally close to zero, when they are estimated relative to average coancestry within each population or continental group but change when the complete set of 26 populations is used as a reference. These latter values compare the allele sharing for each individual to the same reference value, the average sharing over all pairs of individuals in the whole dataset. The world reference gives markedly lower ${\hat{f}}_{{{{{{\rm{AS}}}}}}}$ values for the African populations (AFR), reflecting their higher levels of genetic diversity. The rankings for ${\hat{f}}_{{{{{{\rm{AS}}}}}}}$ within a population, by construction, do not change with reference set. High ${\hat{f}}_{{{{{{\rm{AS}}}}}}}$ values reflect admixture, consanguineous matings and high evolutionary coancestry. In contrast, the ${\hat{f}}_{{{{{{\rm{UNI}}}}}}}$ values are higher for African individuals than for any other individuals when the allele frequencies are from all 26 populations: this reflects an African-specific pattern of negative average individual kinships *ψ*, shown in the bottom row of Fig. 5.

The critical role that average kinship plays in inbreeding estimation is illustrated in Fig. 5. With each reference set, the allele-sharing inbreeding estimates ${\hat{f}}_{{{{{{\rm{AS}}}}}}}$ are clustered for European (EUR) individuals, a little more diverse for East Asian (EAS) individuals, much more diverse for South Asian (SAS) and African (AFR) individuals, and extremely diverse for American (AMR) individuals. These values are consistent with those reported for the numbers of variant sites per genome (The 1000 Genomes Project Consortium, 2015). The variation among African and American average kinships ${\hat{\psi }}_{{{{{{\rm{AS}}}}}}}$ is substantial: as these quantities determine how the expected values of ${\hat{f}}_{{{{{{\rm{UNI}}}}}}}$ and ${\hat{f}}_{{{{{{\rm{STD}}}}}}}$ differ from the *f* target parameters, it is clear that these estimates cannot be used to rank individuals by their inbreeding levels.

For the African population ASW, individual NA20294 has ${\hat{f}}_{{{{{{\rm{AS}}}}}}}$ values of −0.009, 0.001,−0.130 using ASW, AFR or World as a reference set and each estimate is ranked as number 16 among the 61 ASW estimates. The same individual has ${\hat{f}}_{{{{{{\rm{UNI}}}}}}}^{u}$ values of −0.007 (rank 36), 0.001 (rank 16) and 0.028 (rank 60) using ASW, AFR or World allele frequencies. Estimator ${\hat{f}}_{{{{{{\rm{UNI}}}}}}}^{u}$ indicates NA20294 to be among the least inbred of the ASW individuals when AFR sample allele frequencies are used, but among the most inbred when world-wide sample allele frequencies are used, even though the individual’s own genotype is the same for each analysis. Other examples of rankings changing with reference population for ${\hat{f}}_{{{{{{\rm{UNI}}}}}}}$ are shown in Fig. S3; for the admixed ACB and ACB populations, for example, the individuals appearing the most inbred with continental reference appear the least inbred with world reference and vice versa. This can have implications for studies of inbreeding depression, where trait values are regressed on estimated inbreeding coefficients.

**Fig. 6:**
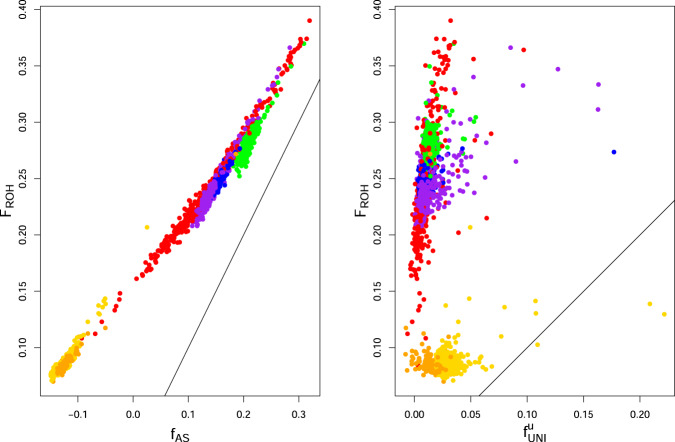
ROH/PLINK estimates vs SNP by SNP estimates for 1000 Genomes data, with the World as a reference set. Left: ${\hat{F}}_{{{{{{\rm{ROH}}}}}}}$ vs ${\hat{f}}_{{{{{{\rm{AS}}}}}}}$; Right: *F*_ROH_ vs ${\hat{f}}_{{{{{{\rm{UNI}}}}}}}^{u}$. Solid line *X* = *Y*. Gold: AFR (not ACB or ASW); Orange: AFR (ACB and ASW); Red: AMR; Purple: SAS; Blue: EUR; Green: EAS.

A comparison of runs-of-homozygosity estimates ${\hat{F}}_{{{{{{{\rm{ROH}}}}}}}_{j}}$ with SNP-by-SNP estimates is shown in Fig. 6. The ROH estimates were produced with the --homozyg --homozyg-snp2 --homozyg-kb100 options in PLINK (Meyermans et al., 2020). The values of ${\hat{F}}_{{{{{{{\rm{ROH}}}}}}}_{j}}$ depend on the PLINK settings for minor allele frequency pruning and linkage disequilibrium pruning, as well as on SNP density, so their expected values may differ from the true *F*_*j*_ values. The left panel shows ${\hat{f}}_{{{{{{{\rm{AS}}}}}}}_{j}}$ values and these have a correlation of 0.998 with ${\hat{F}}_{{{{{{{\rm{ROH}}}}}}}_{j}}$. The right panel shows ${\hat{f}}_{{{{{{{\rm{UNI}}}}}}}_{j}}^{u}$ estimates and these have a correlation of −0.337 with ${\hat{F}}_{{{{{{{\rm{ROH}}}}}}}_{j}}$ estimates.

Gazal et al. (2015) reported inbreeding estimates ${\hat{F}}_{{{{{{{\rm{Fsuite}}}}}}}_{j}}$ from ROH, although their method requires sample allele frequencies and so may have estimates of *F* confounded by average individual-specific average kinships. They also assumed Hardy–Weinberg equilibrium. However, there is good agreement of ${\hat{f}}_{{{{{{{\rm{AS}}}}}}}_{j}}$ values with ${\hat{F}}_{{{{{{{\rm{Fsuite}}}}}}}_{j}}$ values (Fig. S4). The agreement between ${\hat{F}}_{{{{{{{\rm{Fsuite}}}}}}}_{j}}$ and ${\hat{f}}_{{{{{{{\rm{UNI}}}}}}}_{j}}^{u}$ is seen there to be not as good.

## Discussion

Discussions on the estimation of individual inbreeding coefficients generally refer to *F*, the probability an individual has pairs of homologous alleles that are identical by descent. Among the estimators we have considered here, ${\hat{F}}_{{{{{{\rm{ROH}}}}}}}$ addresses *F* by assuming that long runs of homozygous SNPs represent ibd regions. The ROH estimates, however, are conditional on the settings used to calculate the estimates, and actual ibd in short runs of homozygotes may be ignored, so the expected values of these estimators is not known. The Bayesian approach of Vogl et al. (2002) also addresses *F* but at the computational cost of estimating allele proportions in a reference population assumed to have zero inbreeding or relatedness. All the other estimators considered here are, instead, addressing the within-population inbreeding coefficient *f* that compares *F* values to ibd probabilities for pairs of individuals. There is no need to specify the reference population implicit in the definition of identity by descent. There is also no need to assume the particular individuals in a sample have an inbreeding coefficient of zero. For large numbers of SNPs, allele-sharing estimators ${\hat{f}}_{{{{{{\rm{AS}}}}}}}$ are unbiased for *f* for all sample sizes and have values for a set of individuals that have invariant ranks over studies that include that set. We show that most estimators using sample allele frequencies are estimating some combination of *f* and of individual-specific average kinships *ψ* with individuals in the study. Estimators with expectations depending on *ψ* do not have invariant rankings, as we showed with data from the 1000 Genomes project as the study scope varied from the population to the continent to the world.

Our ibd-based model rests on expectations of allele-sharing proportions satisfying expressions such as Eq. (5). There is no requirement for nonoverlapping generations, or homogeneous populations, for example. This generality is a consequence of not needing allele frequencies, whether these refer to a population or to an individual.

The role of ibd probabilities in theoretical population and quantitative genetic contexts is well known, but we suggest it is rank-invariant estimators for the within-population parameters *f*_*j*_ that are of relevance for empirical studies and we offer the examples in the following sections.

### Genotype probabilities

There is often a need to estimate genotype probabilities from observed allele proportions using formulations with allele probabilities and ibd probabilities *F* (e.g., (National Research Council, 1996) for forensic science). Following Eq. (6) we see that it is $2{\tilde{p}}_{l}(1-{\tilde{p}}_{l})(1-{f}_{j})$ rather than $2{\tilde{p}}_{l}(1-{\tilde{p}}_{l})(1-{F}_{j})$ that is unbiased for 2*π*_*l*_(1 − *π*_*l*_)(1 − *F*_*j*_) if *F*_*j*_ and *f*_*j*_ are known.

### Inbreeding depression

Inbreeding is known to affect, linearly, the expected value of quantitative traits, and studies of inbreeding depression often proceed by regressing trait means on inbreeding levels. In Yengo et al. (2017), we used ${\hat{F}}_{{{{{{\rm{ROH}}}}}}}$, ${\hat{f}}_{{{{{{\rm{HOM}}}}}}}$ and ${\hat{f}}_{{{{{{\rm{UNI}}}}}}}$ as inbreeding estimates and Kardos et al. (2018) pointed out that we did not discuss the distinction between *F* and *f*. We responded (Yengo et al., 2018) with reasons for not wishing to use ${\hat{F}}_{{{{{{\rm{ROH}}}}}}}$ and we could have pointed out the linear relationship between *f*_*j*_ and *F*_*j*_ and the high correlation we showed above between ${\hat{f}}_{{{{{{{\rm{AS}}}}}}}_{j}}$ and ${\hat{F}}_{{{{{{{\rm{ROH}}}}}}}_{j}}$ means that regressing on either ${\hat{F}}_{{{{{{\rm{ROH}}}}}}}$ or ${\hat{f}}_{{{{{{\rm{AS}}}}}}}$ should lead to similar results. In less-homogeneous populations than represented by the UK Biobank data (Allen et al., 2012) we used in Yengo et al. (2017), it would appear to be better to use ${\hat{f}}_{{{{{{{\rm{AS}}}}}}}_{j}}$ than ${\hat{f}}_{{{{{{{\rm{UNI}}}}}}}_{j}}$ to avoid any effects of individual-specific average kinships on inbreeding estimates. The correlation of trait and ${\hat{f}}_{{{{{{{\rm{AS}}}}}}}_{j}}$ values is invariant over reference populations. Alemu et al. (2021) pointed out that ${\hat{f}}_{{{{{{\rm{HOM}}}}}}}$ (and ${\hat{f}}_{{{{{{\rm{AS}}}}}}}$), gives equal weights to all SNPs, whereas ${\hat{f}}_{{{{{{\rm{UNI}}}}}}}^{u}$ gives greater weight to SNPs with rare alleles. Alemu et al. did not consider the role of individual average kinships in the bias of ${\hat{f}}_{{{{{{\rm{UNI}}}}}}}$.

### Genetic relatedness matrix

Inbreeding is also known to affect, linearly, the additive component of genetic variance. For additive traits, the genetic variance for individual *j* is $(1+{F}_{j}){\sigma }_{A}^{2}$ where ${\sigma }_{A}^{2}$ is the additive variance for populations in Hardy–Weinberg equilibrium. Consequently, the expected value of the sample variance ${\tilde{V}}_{T}$ of trait values over a sample of *n* individuals is (Speed et al., 2012)

$${{{{{{\mathcal{E}}}}}}}_{T}({\tilde{V}}_{T})=\frac{1}{n}\left({{{{{\rm{tr}}}}}}({{{{{\boldsymbol{G}}}}}})-\frac{1}{n-1}{{{\Sigma }}}_{{{{{{\boldsymbol{G}}}}}}}\right){\sigma }_{A}^{2}+{\sigma }_{e}^{2}$$

Here the trait is additive and the errors, with variance ${\sigma }_{e}^{2}$, are independent of genetic effects. The GRM ***G*** has trace ${{{{{\rm{tr}}}}}}({{{{{\boldsymbol{G}}}}}})$ and sum of off-diagonal elements Σ_***G***_. If the GRM elements are (1 + *F*_*j*_) on the diagonal and $2{\theta }_{jj^{\prime} }$ off the diagonal then the trace is *n*(1 + *F*_*W*_) and the sum of off-diagonal elements is *n*(*n* − 1)*θ*_*S*_ so the genetic component of *V*_*T*_ is $(1+{F}_{W}-2{\theta }_{S}){\sigma }_{A}^{2}$. If the GRM is replaced by a matrix with allele-sharing inbreeding and kinship estimates, this becomes $(1+{f}_{W}){\sigma }_{A}^{2}$, reflecting that it is the within-population estimated GRM that is used in practice. We show elsewhere that the same expected variance holds with GRMs constructed with ${\hat{f}}_{{{{{{\rm{STD}}}}}}}$ or ${\hat{f}}_{{{{{{\rm{UNI}}}}}}}$.

In summary, we have shown that inbreeding measures of utility in empirical studies are “within-population” with the choice of population being at the discretion of the investigator. With allele-sharing inbreeding estimators, the population specifies the set of individuals whose pairwise coancestry is the reference against which inbreeding is measured. For estimators making explicit use of sample allele frequencies, it is the population that furnishes those frequencies, although then inbreeding estimates are confounded by individual-specific average kinships. We showed algebraically and empirically that allele-sharing estimators have invariant rankings across choice of population.

## Software

Estimation of inbreeding coefficients can be performed with the following software.

$${\hat{F}}_{{{{{{\rm{HOM}}}}}}}$$: PLINK

$${\hat{F}}_{{{{{{\rm{Uni}}}}}}}$$: PLINK2, GCTA.

$${\hat{F}}_{{{{{{\rm{Std}}}}}}}$$: PLINK1, GCTA.

${\hat{F}}_{{{{{{\rm{ROH}}}}}}}$: PLINK1, BCFtools/ROH, FSuite.

$${\hat{F}}_{{{{{{\rm{AS}}}}}}}$$: SNPRelate, hierFstat.

$${\hat{F}}_{{{{{{\rm{MLE}}}}}}}$$: SNPRelate.

Software is available at: BCFtools/ROH: https://samtools.github.io/bcftools/howtos/roh-calling.html

FSuite: http://genestat.cephb.fr/software/index.php/FSuite

GCTA: http://gump.qimr.edu.au/gcta

hierFstat:https://cran.r-project.org/web/packages/hierfstat/index.html

PLINK: http://pngu.mgh.harvard.edu/purcell/plink/

PLINK2: https://www.cog-genomics.org/plink/2.0/

SNPRelate:http://www.bioconductor.org/packages/release/bioc/html/SNPRelate.html

## Supplementary information

Simulated pedigree.

Simulated data genoptypes

Gold Coancestries for Simulated Data

Supplementary Figures

## Data Availability

The simulated data are available in the online supplement. The 1000 Genomes data are available at ftp://ftp.1000genomes.ebi.ac.uk/vol1/ftp/.
