# Supplementary material for: Rank-invariant estimation of inbreeding coefficients
Source: Heredity (Edinb). 2021 Nov 25;128(1):1–10. doi: 10.1038/s41437-021-00471-4 (PMC8733021; doi:10.1038/s41437-021-00471-4)
Supplement: Supplementary file 4 — Supplementary Figures [file 41437_2021_471_MOESM4_ESM.pdf]

Supplementary Figure S1: 1000 Genomes Estimates

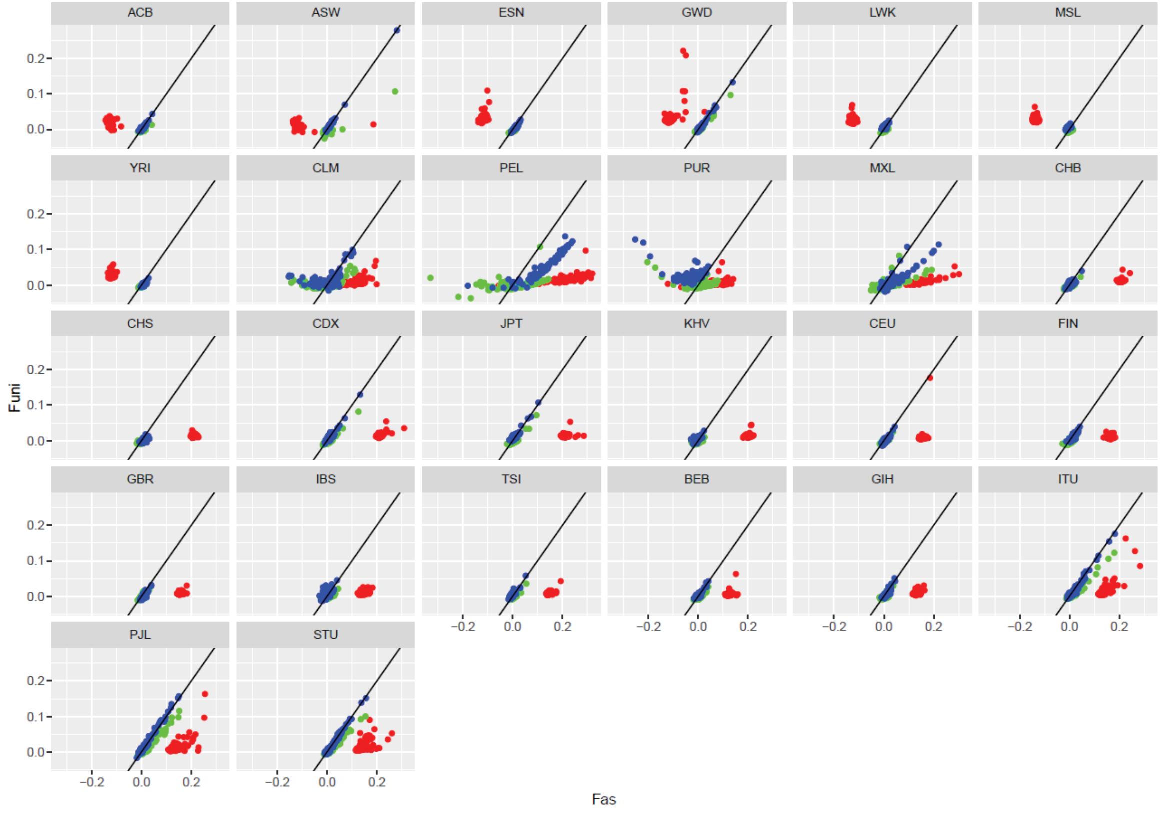

Figure S1: Values of $\hat{f}^u_{\text{UNI}}$ (Y-axis) versus $\hat{f}_{\text{AS}}$ (X-axis) for the 1000 Genomes populations. Population reference in green, continental reference in blue, world reference in red.

Supplementary Figure S2: 1000 Genomes Estimate Ranks

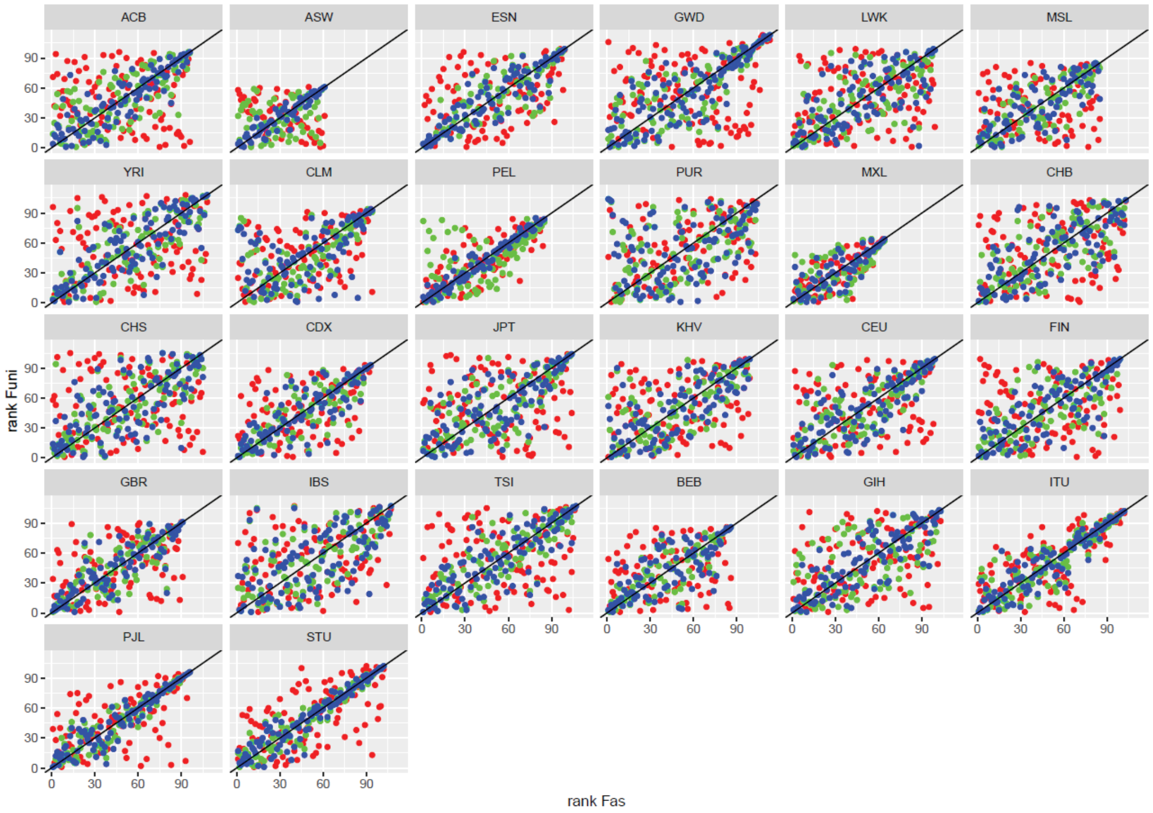

Figure S2: Ranks of $\hat{f}^{u}_{\mathrm{UNI}}$ (Y-axis) versus ranks of $\hat{f}_{\mathrm{AS}}$ (X-axis) for the 1000 Genomes populations. Green: Population reference; Blue: Continental reference; Red: World reference.

Supplementary Figure S3:

Use of Continent vs World Allele Frequencies.

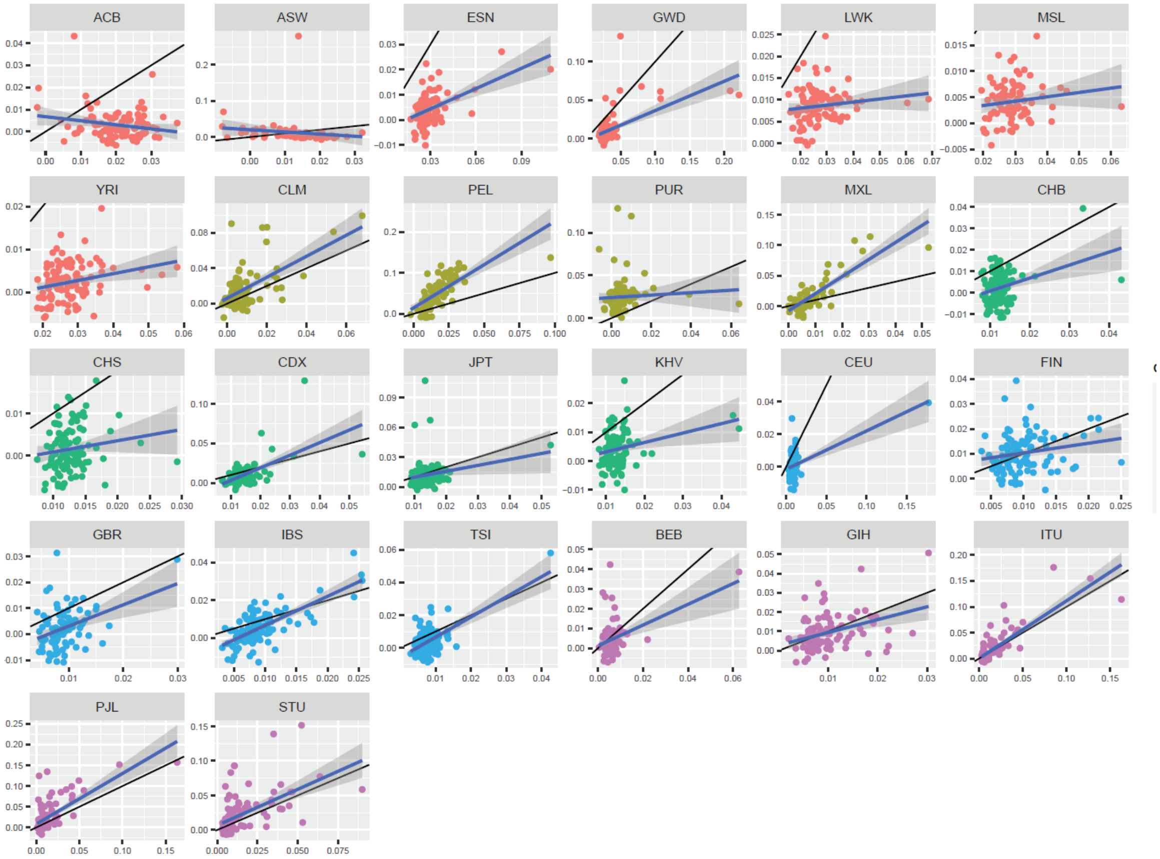

Figure S3: Values of $\hat{f}^{u}_{\mathrm{UNI}}$ for each of the 1000 Genomes populations with the continent for that population providing the sample allele frequencies (Y axis) versus the world providing the sample allele frequencies (X axis). Red: AFR; Gold: AMR; Green: SAS; Blue: EUR; Purple: SAS.

Supplementary Figure S4:

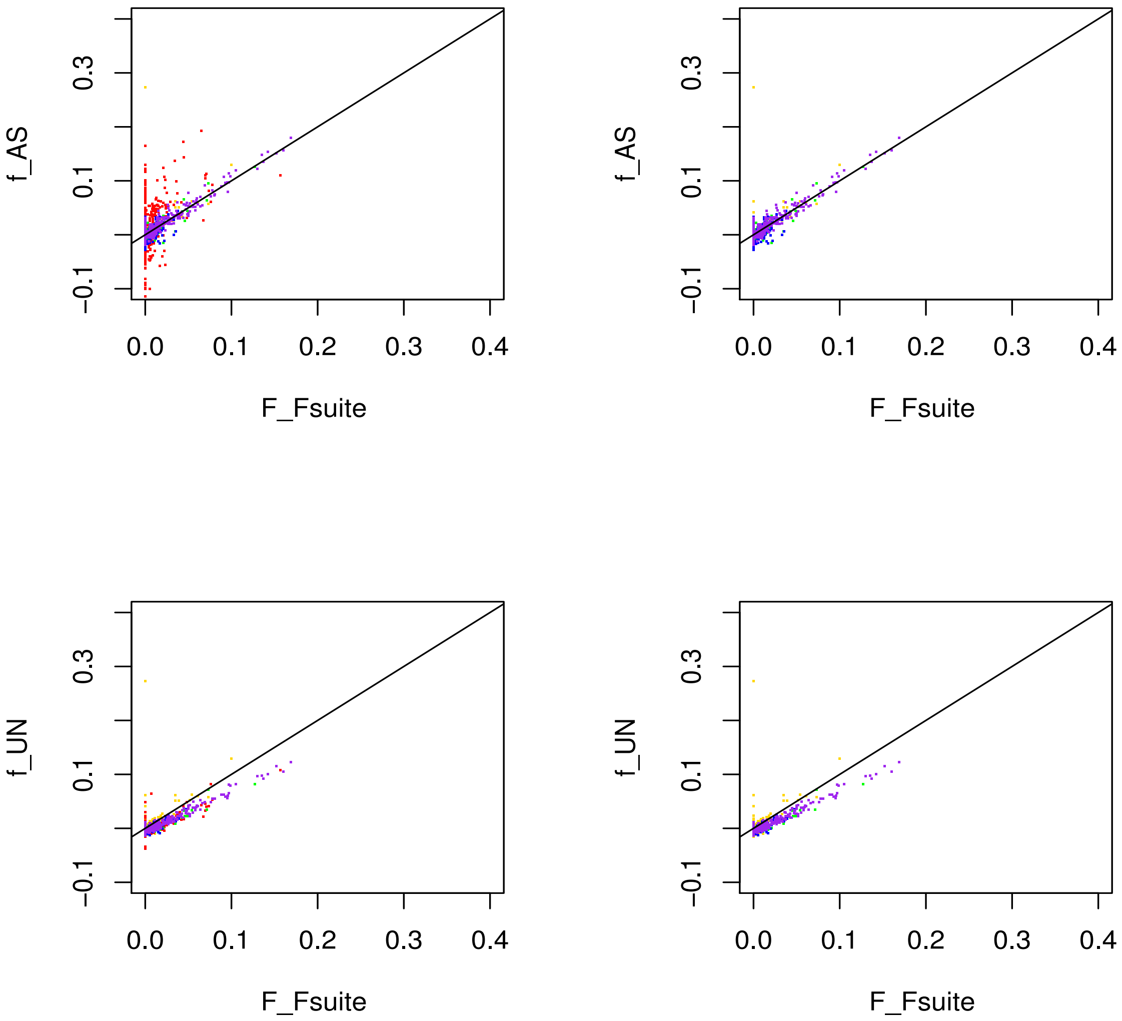

Figure S4: For 1000 Genomes data, with the population as a reference set. Top panels: $\hat{f}_{\text{AS}}$ (Y-axis) vs $\hat{F}_{\text{Fsuite}}$ (X-axis); Bottom panels: $\hat{f}^{u}_{\text{UNI}}$ (Y-axis) vs $\hat{F}_{\text{Fsuite}}$ (X-axis). Left Panels: All 1000 Genomes populations; Right panels: Omitting AMR populations. Solid line $X = Y$ in both panels. Gold: AFR; Red: AMR; Purple: SAS; Blue: EUR; Green: EAS.
